# Tailored intervention for smoking cessation among migrant smokers in health centers for precarious people in Paris: A co-design approach

**DOI:** 10.1016/j.pmedr.2025.103245

**Published:** 2025-09-18

**Authors:** Clair-Antoine Veyrier, Lisa Yombo Kokule, Simon Ducarroz, Caroline Aparicio, Ester Villalonga-Olives, Martin Duracinsky, Lorraine Cousin Cabrolier, Issifou Yaya

**Affiliations:** a Patient-Reported Outcomes Research (PROQOL), Health Economics Clinical Trial Unit (URC- ECO), Hotel-Dieu Hospital, AP-HP, Paris, France; b Université Paris Cité, Inserm, ECEVE, UMR 1123, F-75010 Paris, France; c CNRS, Institut Convergences Migration, Aubervilliers, France; d Policlinique, Hôpital Lariboisière, AP-HP, Paris, France; e Department of Practice, Sciences and Outcomes Research, University of Maryland Baltimore School of Pharmacy, USA; f Medicine Unit, Bicêtre Hospital, AP-HP, Le Kremlin-Bicêtre, France

**Keywords:** Tobacco cessation, Group peer support, Migrants, Co-design, Intervention, France

## Abstract

**Objective:**

Certain groups of migrants in Europe exhibit higher smoking prevalence compared to non-migrants, with social inequalities significantly impacting their health outcomes. **C**ulturally adapted smoking cessation interventions are more effective for migrants. Co-design a smoking cessation intervention tailored to migrant smokers attending in health centers for precarious people in Paris.

**Methods:**

Following an adapted experience-based co-design iterative process to gradually refine our crafted intervention, the study brought together migrants, health professionals, representatives from associations, and a research team between January and July 2024. Pre-workshop enabled to adapt ideation working tools to better suit the public. In the initial phase, migrants helped shape intervention design based on their tobacco use habits and preferences. Subsequent workshops benefitted medical and tobacco-expertise from healthcare workers in co-design workshops, refining our prototypes and ensuring they adhere to evidence-based practices. Data collection included questionnaires, audio-recordings, and field notes analyzed through thematic analysis.

**Results:**

Fourteen migrants (mostly undocumented, from African countries, and current smokers) and fourteen healthcare workers (mostly medical practitioners or nurses) were involved in the co-design sessions. The co-designed intervention consisted in a monthly face-to-face participant-led group discussion supervised by health professionals with flexible attendance combined with WhatsApp group support with facilitated access to nicotine replacement therapy or existing interventions, and adapted health literacy materials.

**Conclusion:**

Co-designing intervention with migrants enabled the development of an intervention addressing their barriers to smoking cessation. Combining peer-support, digital engagement, and facilitated access to existing resources may improve uptake and effectiveness of cessation programs among this vulnerable population.

## Highlights

• Health professionals did not prioritize tobacco cessation for vulnerable migrants.
• WhatsApp with in-person group meetings is very much in demand among migrants.
• Participants valued in person peer group interventions led by health professionals.
• Migrants prefer care outside of their national or ethnic communities.
• Co-design method is relevant to construct an intervention tailored to migrants.

## 1 Introduction

Social inequalities in health steam from the impact of an individual's social position in society, including socioeconomic status, housing, employment, and legal status, on their health outcomes (Lebano et al., 2020; Gazard et al., 2015). Migrants are disproportionately vulnerable population. Migrants tend to be healthier than the host population during the early stages of their migration journey (Newbold, 2005; Razum, 2006). However, over time, disparities, inequalities and health risks inherent to their migrants' status and the migration process begin to emerge. Undocumented migrants are particularly more vulnerable due to their living conditions, making them more susceptible to communicable diseases, including vaccine-preventable illnesses, at later stages (Coppola et al., 2015). Precarious living conditions significantly affect their access to healthcare. In Europe, migrants exhibit a higher smoking prevalence compared to other regions of the world (Amiri, 2020). For instance, in France in 2012, migrants were 31 % more likely to smoke than non-migrants (Solé-Auró et al., 2012). Smoking cessation has substantial health benefits, including reductions in morbidity and mortality from cardiovascular, respiratory, and oncological diseases (Stead et al., 2013; Hurley, 2005). However, the implementation of smoking reduction measures has widened social inequalities in health, particularly regarding smoking behavior, with a higher prevalence of smoking in disadvantaged communities (Frohlich and Abel, 2014).

Despite the establishment of health centers for precarious people (Permanences d'Accès aux Soins de Santé, PASS) in France (Pfister et al., 2014), smoking cessation has not been achieved among migrants using these services (El Chami et al., 2023). The French National Authority for Health (HAS) recommends professional support (face-to-face, by telephone or Internet), nicotine replacement therapy as the first-line treatment for smoking cessation. Vulnerable groups are lacking support from Healthcare professionals (HPs) to quit smoking (Twyman et al., 2014). Tobacco cessation interventions recommended for the general population have not been adequately evaluated among migrants, where cultural factors may play a significant role. Research suggests that culturally adapted interventions — incorporating relevant values, beliefs, and practices — can enhance both the acceptability and effectiveness of smoking cessation efforts (Liu et al., 2013). A tailored intervention targeting Swiss Turkish migrants significantly increased tobacco cessation rates after one year (Castro et al., 2021). Migrants face barriers to quitting smoking, such as a lack of awareness of resources, long work hours, low confidence in cessation services and financial constraints (El Chami et al., 2023; Schnoz et al., 2011; Spence and Zhu, 2017). Beyond cultural barriers, practical barriers prevent migrants from accessing tobacco cessation interventions, lack of information and difficulty in accessing cessation assistance (Mao and Bottorff, 2017).

Public involvement (O'Donnell et al., 2024) can increase the relevance and accessibility of interventions (Twyman et al., 2014). Co-design is a relevant approach to improving health service design (Cosgrave et al., 2022; Jessup et al., 2018; Masterson et al., 2022; Slattery et al., 2020), as it values experiential knowledge to solve problems (Cornish et al., 2023). By involving hard-to-reach populations, co-designed interventions can improve uptake and engagement (Cosgrave et al., 2022). However, involving vulnerable groups in co-design activities often requires some adaptations (Chauhan et al., 2021; Dietrich et al., 2017). This paper outlines the use of an experience-based co-design (Bate and Robert, 2007) approach to develop a tailored smoking cessation intervention for migrants within the Paris area's PASSs.

## 2 Methods

### 2.1 Study design

Relying on empowerment theory (Zamenopoulos et al., 2021), this research employed a three-step sequential design, based on experience-based co-design (Locock et al., 2014), design thinking tools (Altman et al., 2018) and thematic analysis (Chapman et al., 2015). Co-design is grounded in a philosophy of shared power (Zogas et al., 2024) with the active involvement of those affected by the intervention being a key principle of participatory design (Ehn, 2008; Trischler et al., 2019). This study is reported in accordance with the COREQ checklist (Tong et al., 2007).

### 2.2 Setting and participants

The study was conducted from January and July 2024 in three PASSs of the Paris area. We recruited migrants with the following inclusion criteria: adults born in a foreign country, ever-smokers, attended a consultation at a PASS within the last 12 months, and had at least an elementary level of French. The last criteria facilitated the dynamic of co-design workshops. HPs were recruited from PASS who worked with migrant smokers. To maximize recruitment, researchers distributed flyers in PASS waiting rooms, presenting the project. Workshops were conducted at three hospitals fostering participation from a diversity of profiles. Convenient sampling was used. Participants received €30 per workshop, following established practices for engaging hard-to-reach populations (Chauhan et al., 2021). The sample size was not predetermined, and workshops ended when there were no more design problems to discuss or solve.

### 2.3 Workshops details

The research was structured in three phases: 1) preparatory workshop, 2) co-design with migrants, and 3) adaptation and improvement with HPs.

The preparatory workshop established foundational elements for subsequent sessions, gathering key informants: migrants, association representatives, HPs and the research team. Early involvement of participants aimed to enhance participant engagement in later workshops (Zogas et al., 2024) and provided visual resources and interactive activities to foster engagement (Chauhan et al., 2021).

A first series of four two hours co-design workshops were conducted with migrants to develop a prototype intervention with two researchers (CAV, Phd. social sciences accompanied by either LYK, IY, PhD epidemiology or LCC, PhD. Public health). All workshops began with an icebreaker activity using visual aids on participants' experiences, knowledge and cessation attempts. This preliminary stage was intended to “setting the scene” through the definition of a shared understanding of others experiences and a shared challenge facing migrant smokers (Langley et al., 2018). This “sensitizing stage” fosters user engagement and identification with the forthcoming intervention (Dietrich et al., 2017). The sharing of experience was designed as a resource 1) to support the ideation process with migrants, 2) to portray participants for the forthcoming workshop with HPs. The rest of the workshop focused on designing an intervention, with iterative activities of ideation, selection and identification of new “problems”. The initial session defined the core concept, while subsequent sessions refined the prototype through evaluation, and rework on emerging problems and shared priorities for improvement. At the end of each workshop, participants reviewed and approved the selected ideas and shared priorities for further improvement. These outcomes were presented at the following workshop for refinement. Iteration of the workshops ended one the prototype was approved and not further issues emerged.

The subsequent co-design phase included a series of three workshops with various HPs, focusing on refining the intervention prototype initially developed by the migrants. This phase aimed to integrate medical expertise into tobacco cessation practices and provide support for the migrant population within PASS. Each session with HPs was evaluated and refined the intervention iteratively respecting migrants' preferences. Following iterative refinements, the intervention prototype was submitted to the project's scientific committee for endorsement, adhering to the TIDieR checklist standards (Hoffmann et al., 2014).

### 2.4 Data collection

Data collection consisted of a questionnaire on sociodemographic information, audio recordings of each workshop, its verbatim transcription using Noscribe (Dröge, 2024) with manual verification and researcher field notes. Any materials modified or produced during workshops were collected for further refinement between sessions.

### 2.5 Data analysis

At the end of each session, an approved summary of selected ideas and priorities for improvement was made. The progress of the prototype was documented after each workshop. One member of the research team conducted open coding of the transcriptions of each workshop. We employed thematic analysis (Chapman et al., 2015) to focus on users' experiences and preferences, as well as to evaluate the intervention itself. This thematic analysis helped to identify areas that had received less attention during the sessions and provided insights for the workshops with HPs. NVivo 1.7 was used for the analysis.

### 2.6 Ethics

The study was approved by the ethical and regulatory board CER AP-HP Center (IRB ref.: IORG0010044; 12/14/2023). Prior to inclusion, researchers obtained oral consent from each participant. Each workshop began with participants providing written informed consent to participate and permission for the audio recording of the sessions.

## 3 Results

The first two sections examine participant characteristics, experiences and preferences concerning quitting. The next two sections focus on co-design workshops conducted with migrants and HPs. These workshops provide a detailed analysis of how a group intervention for smoking cessation was developed and tailored to meet the needs of migrant smokers. The final section highlights its distinctive features.

### 3.1 Participants' characteristics

Fourteen migrants participated in the workshops, of whom nine (64 %) were undocumented, eight (57 %) were current smokers and one was a woman. Participants came from eight different countries, mostly from African countries. Their characteristics are described in Table 1. Fourteen HPs from three different PASS participated, 3 of them were male (Table 2).

**Table 1:** Characteristics of adult migrants (*N* = 14) who participated in co-design workshops to develop a prototype tobacco cessation intervention tailored to their needs, Paris area, France (January–July 2024).

Characteristics (N = 14)	n	% or mean ± SD
**Age, years**		
*Mean ±* *SD*	14	40 ± 14
**Sex**		
*Female*	1	7
*Male*	13	93
**Country of birth**		
*Algeria*	1	7
*Cuba*	1	7
*Ivory Coast*	4	29
*Guinea*	2	14
*Mali*	2	14
*DRC*	1	7
*Senegal*	2	14
*Tunisia*	1	7
**Smoking status**		
*Current smoker*	8	57
*Former smoker*	6	43
**Smoking duration, years**	8	21 ± 14
**Duration of smoking cessation, years**	6	6 ± 5
**Tobacco product**		
*Cigarette*	12	86
*Rolling tobacco*	2	14
*Cigarillos*	1	7
**Administrative status**		
*Asylum seeker*	2	14
*Regular resident*	3	21
*Undocumented*	9	65

**Table 2:** Characteristics of healthcare professionals (N = 14) working in health centers for precarious people who participated in co-design workshops to develop a prototype tobacco cessation intervention for migrants, Paris area, France (January–July 2024).

Characteristics (N = 14)	n	% or mean ± SD
**Age, years**		
*Mean ±* *SD*	14	43 ± 10
**Sex**		
*Female*	11	79
*Male*	3	21
**Professional category**		
*Nursing Assistant*	1	7
*Social worker*	1	7
*Nurse*	5	*36*
*Resident physician*	1	7
*Practitioner*	6	43
**Professional experience, years**	14	15 ± 11
**Training on tobacco cessation**		
*No*	13	93
*Yes*	1	7

Only one participant had specific training in tobacco cessation. Most of them were either physicians (Amiri, 2020) or nurses (Coppola et al., 2015).

### 3.2 Participant experiences and preferences about tobacco cessation

Most participants were current cigarette smokers, with significant variation in their consumption patterns over time. Smoking often served as a coping mechanism to mitigate stress and loneliness. Few were actively seeking information on quitting. Their knowledge varied, with only one participant mentioning online research. Some participants were unaware of nicotine substitutes or expressed hesitation about using them.

“There are a lot of people who want to quit, but they can't find a platform to apply.” (F10, Male, ≈40 years).^1^

Former smokers had quit smoking without medical assistance: “you have to decide, that's it, it's over” (F04, female, former smoker) They often used willpower and strong desire to quite as a shared representation.

“Maybe it's only the individual's determination that can get him through. I think that's it. Really some firm resolve.” (F13).

Participants emphasized the role of the environment in quitting smoking or staying motivated. They believed that modifying their environment was essential for smoking cessation.

“it helps the mind focus on something other than cigarettes” (F10, Male, ≈40 years).

Some participants hid their smoking habits from HPs. Social isolation made it difficult to discuss quitting. Most participants wanted assistance from HPs; however, not all received brief advice or support. Many perceived brief advice as coercive rather than supportive. Most HPs in the workshops reported routinely asking about tobacco use and providing brief advice. One professional acknowledged treating migrants differently regarding tobacco cessation:

“When they tell us they smoke, as they have very complicated life situations, we say to ourselves, well … they smoke because life's no fun and that's that. So, we're not going to take it seriously… It's a problem that we're not going to address in the same way.” (P11, male, physician).

Those shared experiences were a basis for discussion to codesign an adapted intervention.

### 3.3 Workshop with end users: toward a group intervention

**Fig. 1:**
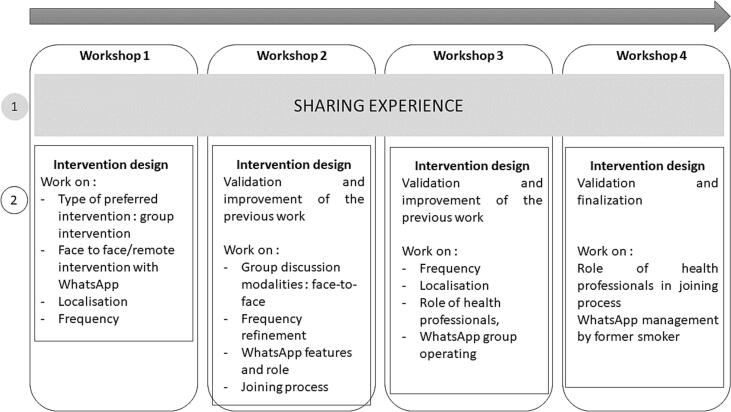
Key steps and problems discussed with migrants to design a prototype ttobacco cessation intervention tailored to their needs, Paris area, France (January–July 2024).

Four workshops were held with fourteen migrants for a total of 7 h and 20 min (between 90 min and 140 min each session). Fig. 1 summarizes the main steps and problems discussed during each workshop with the participants.

The ideation process converged with the concept of a group support meeting for tobacco cessation, bringing together current or former smokers to combat isolation faced by migrants. The participants also discussed the potential formats of such meetings.

“You're already isolated when you smoke, so to be taken care of alone again [is not a preference]” (F01, Male, ≈50 years).

This initiative offers a platform for people to discuss their experiences of quitting tobacco. They envisioned this group as a place to share tips and tricks for quitting tobacco, to discuss nicotine substitutes and addiction mechanisms, and to provide mutual support and “set goals for each other” (F01, Male, ≈50 years):

“Willpower can only be efficient when it is supported, in my case at least, by an environment that acts as a sort of safeguard” (F10, Male, ≈40 years).

This aligns with their view of the environment as for quitting smoking. Participants confirmed that the composition of groups should not be limited to specific nationalities or common medical issues beyond the common goal of quitting tobacco:

“You have to mix and match, because health has no borders, no colors, no class. So, exchanging with a Tunisian, an Algerian from his experience and me an Ivorian from my experience I think can help the group.” (F08, Male, ≈55 years).

This preference was shared across all group sessions. They suggested that relatives could occasionally attend meetings. This group composition preference raises the question of language barriers. Diversity in groups was seen as a force, since they speak different languages and could help each other. As a participant explained:

“When intentions are shared, language no longer becomes a barrier.” (F10, Male, ≈40 years).

In the second workshop, participants preferred face-to-face group interventions over remote sessions. They recommended reducing the frequency to once per month, with a clear schedule and a designated facilitator managing registrations. Localization was not considered a priority. They also proposed using WhatsApp for group sessions. The purpose was to enhance accessibility, maintain motivation, engage participants and disseminate organizational updates, reminders and evidence-based cessation strategies. Participants preferred using WhatsApp, a common platform, as a convenient way to stay in touch with both pairs and HPs:

“I'd prefer us to talk more about tobacco mechanisms and tips for helping each other [on WhatsApp].” (F08, Male, ≈55 years).

Participants suggested that a former smoker could manage the WhatsApp group and organize special remote events with HPs. Invitations to join the group session will be provided by HPs. Information on the group intervention would include a flyer with a QR code for accessing a contact form. WhatsApp was seen both as a support tool and as a way of recruiting new participants more easily. They also suggested group members to directly enroll other participants in the WhatsApp group.

### 3.4 Co-design workshops with health professionals

**Fig. 2:**
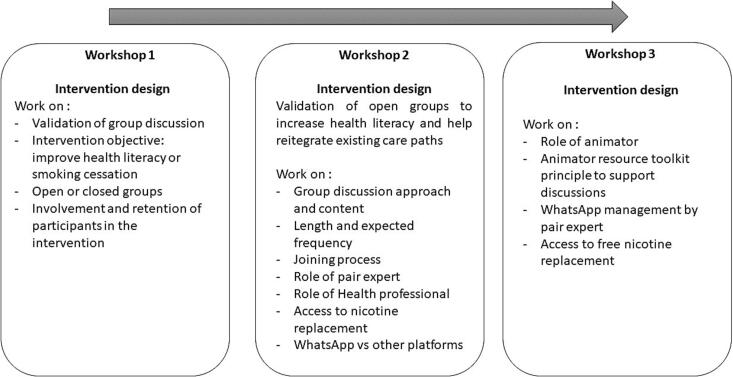
Key steps and problems discussed with health professionals to design a prototype ttobacco cessation intervention tailored to their needs, Paris area, France (January–July 2024).

Three co-designed workshops were held with fourteen HPs, totaling six hours (Between 104 min and 257 min. Per session). Fig. 2 presents the main different steps and topics discussed during each workshop with HPs.

Two proposals emerged. First a tight-knit group of participants followed for 3 to 6 months, with each session setting specific goals toward quitting smoking, being all sessions mandatory. However, HP underlined the limit of closed groups with this population:

“The problem with the closed group is that I don't think we can be sure that people are really coming very regularly […] So when we offer this type of thing, they like it when we tell them that they don't have to come.” (P06, female, physician).

The initial proposal might yield greater advancement for individuals, but it could limit its reach. The second suggestion was to create a concise program aimed at enhancing migrants' understanding of tobacco cessation, while integrating current support networks to help them quit. Tobacco-related health literacy emerged as a crucial aspect that could benefit more people. HPs expected to retain migrants for three months, the duration migrants usually visit a specific PASS. They prioritized participant-driven discussion to avoid a top-down approach.

“We all know that top-down literacy doesn't work very well.” (P12, Female, physician).

This choice led to the idea of a toolkit with resources on tobacco cessation, nicotine substitutes, tobacco addiction mechanisms, motivation, relying on adapted or existing supports in PASS.

“It's good that there's time for information, that it's not locked in beforehand, that it adapts to what the group wants.” (P12, Female Physician).

Health literacy should be seamlessly incorporated into:

“take-home messages” at the end of each session so that “it fits harmoniously with the idea of a discussion group” (P05, female tobacco expert).

To improve access to existing tobacco cessation resources, such as nicotine substitutes, HPs could offer participants access to these resources during the sessions. This could increase participation and support smoking cessation efforts.

“It's very simple. It's a discussion group. It's pretty vague for people. Maybe it makes them want to know what it is (…) And there is a small reward that motivates me: I'm going to get something at the end, even if it hasn't done me any good.” (P02, female, physician).

Organizing sessions at the hospital would make it easier for participants to access free nicotine substitutes, as the target population typically does not have insurance coverage for these treatments.

### 3.5 Resulting co-designed intervention characteristics

The co-design sessions established a framework for developing a smoking cessation program tailored to Parisian PASS's migrant smokers. The objective of the co-designed program is to enhance health literacy on tobacco cessation and to boost the quit rate among migrant smokers over a 3-month period. The intervention includes in-person group discussions, WhatsApp support, and facilitated access to nicotine replacement therapy.

Cessation group meetings will consist of five to ten migrant smokers, former smokers, relatives (occasionally) and two HPs. The group sessions will take place in person in a PASS at least once a month, with additional sessions if necessary. The schedules will be regular and predetermined. The meeting will last approximately two hours, conveniently scheduled during the pharmacy's operating hours, to make nicotine replacement therapies more accessible. Individuals are invited to attend at any time, promoting attendance and inclusivity. Participation is encouraged for three months, but attendance is flexible. At each meeting, newcomers will have the opportunity to introduce themselves, set goals, discuss concerns, and ask questions. Discussions are participant-driven to avoid a top-down approach. However, a thematic toolkit for group sessions stocked with resources on nicotine substitutes, tobacco addiction mechanisms, motivation explained in plain language will be provided to animators to support discussion with participants.

The co-designed intervention should include a WhatsApp to supplement the in-person sessions. An expert patient would oversee the community, moderating discussions, providing meeting details, predetermined materials and resources on smoking cessation. The WhatsApp group will serve as a valuable platform for participants to share their concerns, exchange tips and strategies, and provide mutual support to maintain motivation throughout their tobacco cessation journey.

The intervention should involve at least two HPs conducting sessions, an expert patient to lead the group and a resource contact from PASS. They must be trained in tobacco cessation techniques and have ability. It is recommended that an expert patient manages the WhatsApp group and maintains contact with the PASS resource person.

Potential participants should be identified through consultations at PASS, where HPs inquire about smoking habits and direct them to the appropriate program. Adapted flyers to migrant health literacy in waiting rooms would promote group sessions and encourage discussions on tobacco cessation with HPs.

## 4 Discussion

The aim of this paper was to outline the process of constructing a smoking cessation intervention tailored for migrants developed through an adapted experienced based co-design approach. A primary challenge faced in this context is the lack of tailored programs for migrants (Schnoz et al., 2011). We co-designed an intervention that combined face-to-face group discussions, WhatsApp support, adapted flyers to migrant health literacy and facilitated access to nicotine replacement therapies. The preferred intervention method aligns with literature suggesting that peer support increases smoking abstinence (Yuan et al., 2023) and text messaging interventions promote quitting (Puljević et al., 2024).

Our results suggest that the participants' migrants were not actively seeking information concerning tobacco cessation. A previous study in a similar context found that this population lacked knowledge of available resources (El Chami et al., 2023). A study among the Chinese community in Great Britain underscored the significance of enhancing informational accessibility to increase uptake of smoking cessation services (Spence and Zhu, 2017). During our design workshops, we discussed how HPs address tobacco issues for migrants in precarious conditions. Some HPs didn't prioritize this, and one even acknowledged treating migrants differently. This lack of support by HPs is shared among vulnerable populations (Twyman et al., 2014). Among cancer patients, asking for smoking is not followed by action by HPs (Frazer et al., 2025). A few participants reported being prescribed nicotine replacement therapy. This aligns with a study in the USA that found that migrants were less likely to receive nicotine replacement therapy (Chen et al., 2020), and another study in California showed Latino smokers received less advice than non-Latino whites (Valencia et al., 2022).

Barriers to quitting smoking include a lack of information about resources (El Chami et al., 2023), long working hours, low confidence in cessation services, cultural barriers, and unsuitable services (Spence and Zhu, 2017), financial concerns and language barriers (Schnoz et al., 2011; Spence and Zhu, 2017). Existing interventions often focus on specific national or ethnic groups (Schnoz et al., 2011; Spence and Zhu, 2017) stressing that migrants are not a homogeneous group (O'Donnell et al., 2024) but our research reveals that migrants with a common smoking habit and visiting PASS facilities prefer care outside of their national or ethnic communities. This preference implies that a shared given language will be chosen for the intervention. After implementing and evaluating the initial co-designed intervention in French, further consideration could be given to interventions in other shared languages.

Participants valued group interventions, which helped lift them out of their feelings of isolation. A review emphasized the importance of social support for smoking cessation among disadvantaged populations (Ford et al., 2013), with migrants often lacking such opportunities (Ford et al., 2013). The preferred support was peer support supervised by HPs. Peer support has been proven to enhance smoking abstinence (Yuan et al., 2023). It entails the exchange of experiences and offers social and emotional support to group members (Manning et al., 2020). A Swiss study underscored the importance of robust relationships in migrant groups for tobacco cessation (Schnoz et al., 2011).

Peer support was designed to be conducted in person, but we've found that using WhatsApp alongside could enhance engagement and support (Cheung et al., 2023). This platform is widely favored among migrants (Calvo et al., 2024), regardless of age (Jauhiainen and Tedeschi, 2021) or gender (Merisalo and Jauhiainen, 2021). Online group discussions have been shown to increase abstinence in quitters (Cheung et al., 2015; Mersha et al., 2024) and text messaging-based interventions have been proven effective (Puljević et al., 2024). However, online group discussions may not be more effective than simple personalized prompts (He et al., 2024).

This intervention would connect migrant populations to existing tobacco cessation resources, enhancing their health literacy (El Chami et al., 2023). Health disparities and limited access to quality healthcare are common among migrants (Ward et al., 2019). We facilitated entry through PASS introductions, informational flyers adapted to migrants' health literacy, and flexible group attendance. Free nicotine replacement therapy addressed financial constraints (Spence and Zhu, 2017). WhatsApp reminders would tackle absenteeism (Pires et al., 2024). Multiple appointment schedules related to their administrative status (Karine and Cretton, 2021) and working hours were identified as potential hindrances to attendance; thus, we combined regular session occurrence with flexibility in attendance.

Working with vulnerable populations in co-design activities requires some adaptations (Chauhan et al., 2021; Dietrich et al., 2017) following core principles instead of a set of rigid steps (Moll et al., 2020). We employed a modified version of the co-design method to engage with vulnerable migrant communities. To address differences and varying ability to participate (Moll et al., 2020), we involved different participants for each session and tailored the recruiting process to increase attendance. We strategically separated HPs from migrant participants during distinct design phases to foster an environment where lived experiences could be openly shared (Zogas et al., 2024). The thematic analysis of transcribed sessions helped to give greater visibility to participants' lived experiences. The findings may not be representative of the experiences and views of all migrants attending PASSs and HPs. We faced difficulties recruiting migrant female smokers, and migrant men were overrepresented, a limitation to the tailored intervention. The language barrier (Schnoz et al., 2011; Spence and Zhu, 2017) may remain because the intervention does not cater to a specific migrant community. Co-design's participatory approach allows for self-selection of engaged participants, enhancing the intervention's relevance and acceptance among the target audience. However, it limits its generalizability.

## 5 Conclusions

Migrants have limited access to preventive and essential care (Gazard et al., 2015) and are less likely to be addressed prescribed nicotine replacement therapy. Different barriers prevent migrants from attending tobacco cessation programs. Co-design was relevant to improve uptake and engagement in the difficult to reach population and propose a tailored intervention, facilitating connecting this population to existing resources and enhancing their health literacy.

## CRediT authorship contribution statement

**Clair-Antoine Veyrier:** Writing – review & editing, Writing – original draft, Software, Methodology, Formal analysis, Data curation, Investigation. **Lisa Yombo Kokule:** Writing – review & editing, Conceptualization. **Simon Ducarroz:** Writing – review & editing, Conceptualization. **Caroline Aparicio:** Writing – review & editing, Conceptualization. **Ester Villalonga-Olives:** Conceptualization, Writing – review & editing. **Martin Duracinsky:** Writing – review & editing, Conceptualization. **Lorraine Cousin Cabrolier:** Writing – review & editing, Methodology, Conceptualization, Investigation. **Issifou Yaya:** Writing – review & editing, Project administration, Investigation, Conceptualization, Supervision.

## Funding

Funding for this study was provided by the National Cancer Institute of France (INCa, N°INCA_16464). The funder did not and will not have a role in study design, data collection and analysis, decision to publish, or preparation of the manuscript.

## Declaration of competing interest

The authors declare that they have no known competing financial interests or personal relationships that could have appeared to influence the work reported in this paper.

## Data Availability

The authors do not have permission to share data.

## Appendix A Supplementary data

Supplementary material: COREQ (COnsolidated criteria for REporting Qualitative research) Checklist. Image 1

## Footnotes

1 All citations are translated from French by the authors.
